# Enhanced Toxicity, Physiological Disruption, and Population Growth Suppression Induced by Nanoemulsified *Satureja hortensis* Essential Oil on *Spodoptera frugiperda*

**DOI:** 10.3390/plants15111598

**Published:** 2026-05-22

**Authors:** Zahra Afrazeh, Marziyeh Oftadeh, Azim Nemati, Jalal Jalali Sendi, Asgar Ebadollahi, William N. Setzer

**Affiliations:** 1 Department of Plant Protection, Faculty of Agricultural Sciences, University of Guilan, Rasht 4199613776, Iran; afrazeh.fereshteh73@gmail.com (Z.A.); marziye.oftade@gmail.com (M.O.); azim.nemati@gmail.com (A.N.); jjalali@guilan.ac.ir (J.J.S.); 2 Department of Plant Sciences, Moghan College of Agriculture and Natural Resources, University of Mohaghegh Ardabili, Ardabil 5697194781, Iran; 3 Department of Chemistry, University of Alabama in Huntsville, Huntsville, AL 35899, USA; 4 Aromatic Plant Research Center, 230 N 1200 E, Suite 100, Lehi, UT 84043, USA

**Keywords:** fall armyworm, botanical insecticide, biochemical assay, detoxification, life table

## Abstract

Although the effectiveness of plant-derived essential oils (EOs) against several insect pests is well-documented, their high volatility presents a challenge. In this study, the potential to enhance the insecticidal activity of *Satureja hortensis* L. EO, an accessible natural agent, through nanoemulsification was assessed against the cosmopolitan pest *Spodoptera frugiperda* (J. E. Smith, 1797). The nanoemulsion of the EO (NEEO) was prepared using Tween 80 as the emulsifying agent and high-intensity ultrasonication. Oral bioassays indicated that the NEEO was more toxic (LC_50_ = 0.922%) than the pure EO (LC_50_ = 1.186%). Sublethal exposure to LC_30_ of the NEEO caused evident reductions in preadult survival, developmental time, fecundity, and oviposition period, as well as the population growth parameter net reproductive rate (*R*_0_). The exposure to the NEEO increased catalase (CAT), glutathione *S*-transferase (GST), and superoxide dismutase (SOD) actions and inhibited α-esterase (α-NE), β-esterase (β-NE), and cytochrome P450 (CYP450) actions. Both the NEEO and EO inhibited acetylcholinesterase (AChE) and Na^+^/K^+^-ATPase, with higher inhibition in the NEEO group. Generally, *S. hortensis* NEEO enhanced toxicity, intensified physiological perturbations, and caused greater negative impacts on population growth parameters. Consequently, nanoemulsification of *S. hortensis* EO can be considered an effective method to strengthen the insecticidal potential of this natural agent.

## 1. Introduction

The fall armyworm, *Spodoptera frugiperda* (J. E. Smith, 1797), is a polyphagous moth species that causes significant economic losses by feeding on more than 350 plant species [1]. Affected crops include essential foods like maize, sorghum, rice, and soybeans, as well as industrial crops such as cotton [2]. The pest comes from tropical and subtropical regions of the Americas, and outside its native range, was first reported in West Africa in early 2016 [3]. It quickly spread throughout sub-Saharan Africa, reaching more than 109 countries by 2022, and later expanded into India, China, and other nearby regions [3,4]. In October 2023, *S. frugiperda* was found for the first time in Iran, specifically in the Orzuiyeh region of Kerman Province [5]. During 2016, 17 *S. frugiperda* outbreaks occurred, and many governments provided farmers with free pesticides and equipment, but these efforts were not enough [6,7]. The rapid reproduction, migratory habits, and adaptability of the pest, as well as the quick onset of insecticide resistance among exposed populations, have challenged control strategies [8]. For instance, in Brazil, Carvalho et al. found that *S. frugiperda* showed 18 to 28 times more resistance to organophosphates and pyrethroids compared to non-resistant populations, allowing for significant population growth [9].

Botanical insecticides are considered a promising option for pest management, particularly due to their potential to control pest populations [10,11,12]. In line with the principles of integrated pest management (IPM), the development of eco-friendly pest control tools based on plant-derived compounds represents an important step toward reducing dependence on conventional synthetic insecticides and promoting more sustainable agricultural practices [13]. Unlike synthetic pesticides, botanical insecticides such as EOs are generally considered safer since they decompose quickly, leave little residue, and are less likely to harm humans and non-target organisms [14]. Their multiple modes of action against target pests can also decrease the resistance issue [15,16]. However, EOs come with limitations, including low water solubility, rapid degradation, and high volatility, which make their direct application in the field difficult. So, developing stable and efficient delivery systems is crucial to improve their effectiveness. In this context, NEEOs have become an important strategy, as they create spherical nanodroplets by mixing immiscible liquids, like EOs and water, with an appropriate emulsifying agent to lower interfacial tension. This method allows for more efficient application of EOs, making them safe alternatives to conventional synthetic pesticides [17].

Summer savory, *Satureja hortensis* L., is one of the most popular species from the Lamiaceae family, which is cultivated in many countries worldwide [18]. Its leaves and stems are commonly used fresh or dried as seasoning and in traditional medicine, particularly for digestive and respiratory purposes [18,19]. Previous studies confirm that *S. hortensis* EO, enriched by terpenenic compounds like carvacrol, γ-terpinene, and thymol, exhibits potent insecticidal and acaricidal activity against agricultural pests [20,21,22,23].

It was revealed that EOs have multiple modes of action against insect pests [14]. EOs can induce neurotoxic effects in insects, leading to behavioral symptoms similar to those caused by organophosphate insecticides [24]. Detoxifying enzymes play a substantial role in helping insects neutralize plant-derived chemicals, called xenobiotics, by removing harmful substances through different detoxification pathways [25,26,27]. The activation of carboxylesterases (CarEs), mixed function oxidases (MFOs), and glutathione *S*-transferases (GSTs) is a crucial defense mechanism. These enzymes are necessary for breaking down plant extracts and EOs by improving their water solubility, causing their swift removal from the insect’s body [28,29]. This enzymatic response enables insects to survive exposure to potentially harmful plant chemicals. In addition, physiological adjustments in insect pests may result in longer developmental periods, shorter adult lifespans, and reduced reproductive capacity across generations [30,31].

Therefore, this study aims to assess not only direct mortality but also the sublethal effects of *S. hortensis* and its nanoformulations, providing useful insights into their potential as biopesticides for integrated pest management (IPM) strategies targeting *S. frugiperda*. These effects are best evaluated through demographic toxicology approaches, using life tables and population projection models to measure long-term impacts on pest populations. Key metrics include developmental delays, reproductive suppression, and population growth inhibition, all crucial for predicting the real-world effectiveness of sustainable pest control systems.

## 2. Results

### 2.1. Chemical Profile of the EO

The *S. hortensis* EO was analyzed by GC-MS, which allowed for identification of 38 components (96.1% of the total composition (Table 1)). The main compounds in the EO were carvacrol (24.0%), γ-terpinene (21.3%), *p*-cymene (19.2%), and α-pinene (6.0%).

### 2.2. Morphological Characterization of S. hortensis NEEO

**Figure 1:**
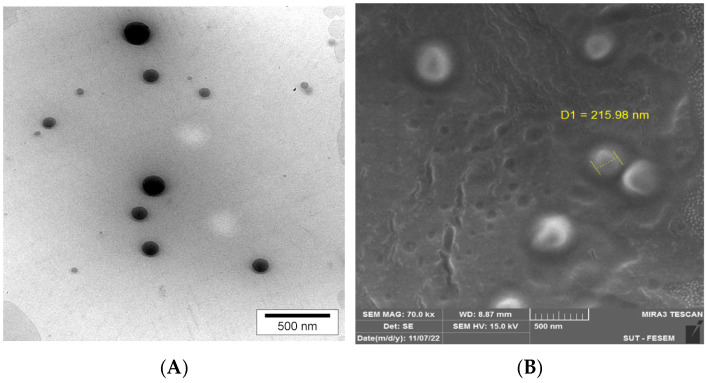
Transmission electron micrographs (TEMs) (**A**) and scanning electron microscopy (SEM) image (**B**) of the nanoemulsion of *Satureja hortensis* essential oil.

The morphological structure of the *S. hortensis* NEEO was clearly evident in microscopic analyses. According to the TEM image (Figure 1A), the synthesized nanoparticles were well-dispersed, spherical, and exhibited smooth, uniform surfaces, predominantly within a size range <250 nm. SEM analysis of the particle surfaces (Figure 1B) further confirmed this spherical morphology, indicating the absence of aggregation and demonstrating the stability of the droplets throughout the NEEO preparation process.

### 2.3. Toxicity

Results revealed a clear concentration-dependent mortality of the second-instar larvae of *S. frugiperda* exposed to *S. hortensis* EO and its NEEO. Both treatments induced dose-dependent larval mortality, although the NEEO exhibited significantly higher toxicity compared to the unformulated EO. The calculated LC_30_ and LC_50_ values for *S. hortensis* EO were 0.743% and 1.186% (*w*/*v*), respectively, while these values for the NEEO were lower: 0.577% and 0.922% (*w*/*v*), indicating an increase in insecticidal potency (Table 2).

### 2.4. Life Table Study

Developmental durations of *S. frugiperda* varied significantly among treatments: see the LC_30_ values of the EO, NEEO, and control groups (Table 3). Egg incubation period was shorter in the NEEO treatment (1.87 ± 0.05) compared with the EO (2.18 ± 0.06) and control (2.26 ± 0.07). In both treatments, the development was accelerated in the first–third and fifth–sixth instar larvae, in which the LC_30_ of the NEEO had the strongest effect. No significant difference occurred in the fourth instar or prepupal stage. Pupal duration was significantly shorter in the EO (7.48 ± 0.09) and NEEO treatments (7.52 ± 0.10) than in the control (9.03 ± 0.11).

Total preadult duration was reduced from 29.74 ± 0.26 (control) to 24.26 ± 0.41 (NEEO), with EO yielding intermediate results. The preadult survival rate decreased from 0.85 ± 0.05 for the control to 0.72 ± 0.07 and 0.57 ± 0.07 for the EO and NEEO, respectively (Table 3).

Adult longevity decreased significantly in both sexes under sublethal exposure. Females lived for 9.56 ± 0.12 in the control group and 8.07 ± 0.16 and 7.69 ± 0.13 in the EO and NEEO treatments, respectively; males showed the same trend (shortest in LC_30_ nano: 6.70 ± 0.21). Thus, total lifespan was reduced significantly in treated cohorts (Table 3).

The adult pre-oviposition period (APOP) was prolonged slightly, while the total pre-oviposition period (TPOP) was reduced under both treatments. Fecundity decreased drastically in the EO and NEEO groups. The oviposition period was also shortened from 6.61 ± 0.12 to 4.64 ± 0.19 and 4.38 ± 0.18 for the control group and EO and NEEO treatments, respectively (Table 3).

**Figure 2:**
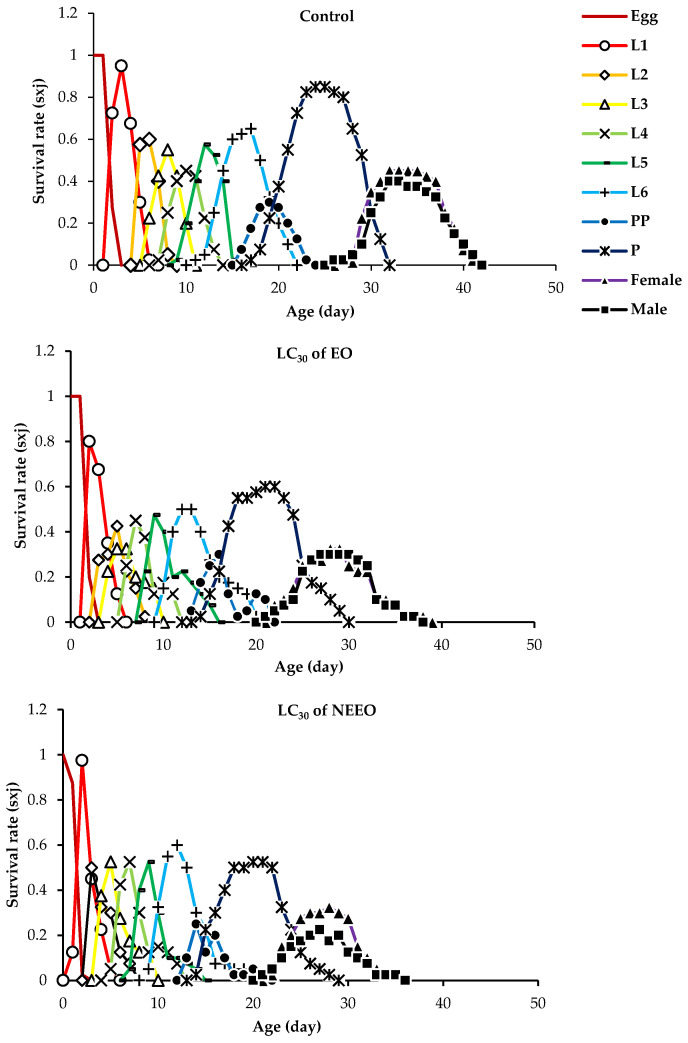
Age-stage-specific survival rate (*sxj*) of *Spodoptera frugiperda* treated with *Satureja hortensis* essential oil (EO) and its nanoemulsion (NEEO), along with a control group.

**Figure 3:**
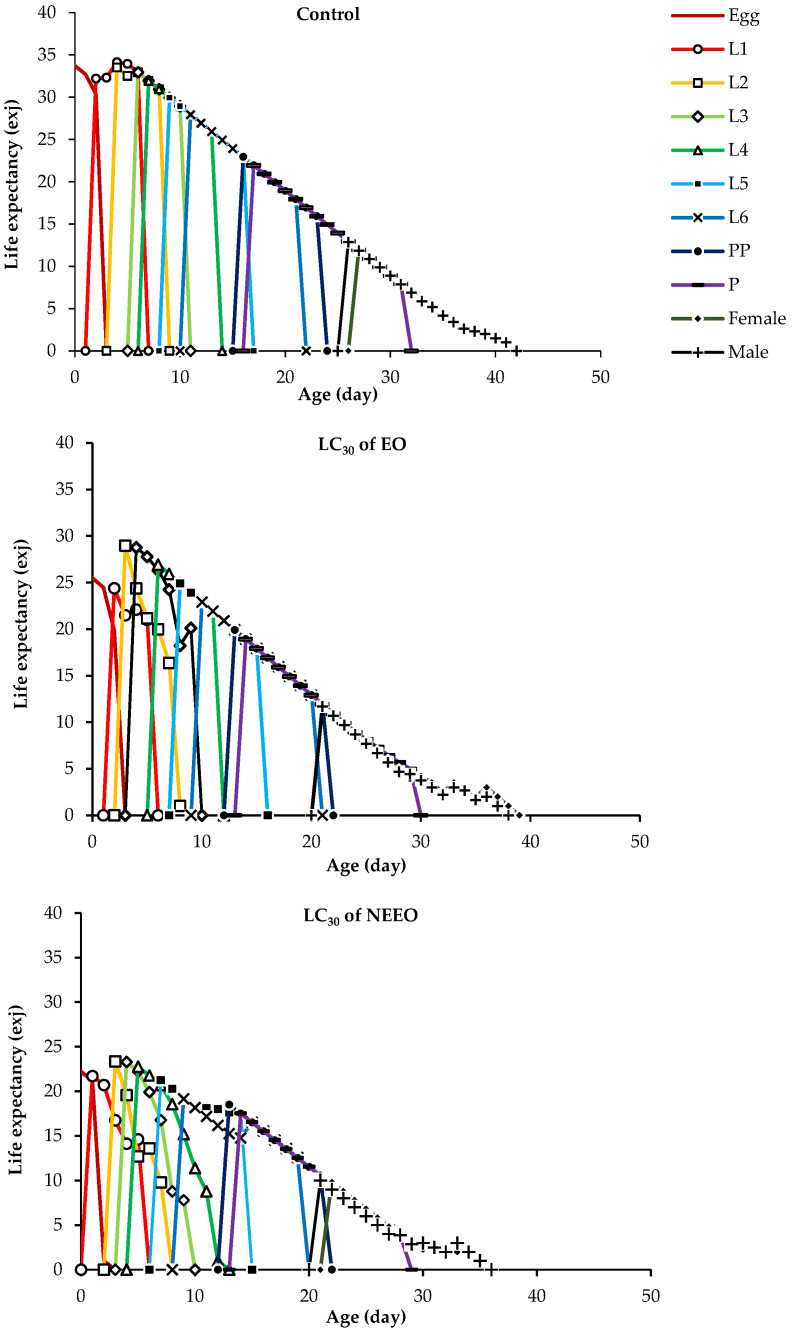
Age-stage-specific life expectancy (*e_xj_*) of *Spodoptera frugiperda* treated with *Satureja hortensis* essential oil (EO) and its nanoemulsion (NEEO), along with a control group.

Age-stage-specific survival rate (*s_xj_*) and life expectancy (*e_xj_*) of the insect pests are shown in Figure 2 and Figure 3. The survival rate and life expectancy were highest in early instars and decreased progressively.

**Figure 4:**
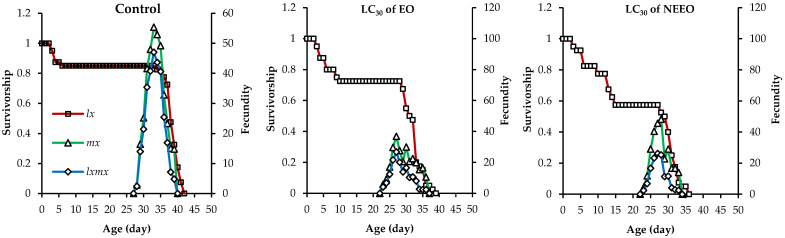
Age-specific survivorship (*lx*), age-specific fecundity (*mx*), and age-specific maternity (*lxmx*) of *Spodoptera frugiperda* treated with *Satureja hortensis* essential oil (EO) and its nanoemulsion (NEEO), along with a control group.

Age-specific survivorship (*l_x_*), fecundity (*m_x_*), and maternity (*l_x_m_x_*) confirmed that peak reproductive contribution occurred shortly after adult emergence (Figure 4).

Population parameters were differentially affected. Gross reproductive rate (*GRR*) did not exhibit any significant differences, though numerically higher in nanoformulation treatment. The net reproductive rate (*R*_0_) significantly decreased from 299.51 ± 52.59 offspring/individuals in the control group to 158.25 ± 34.58 and 133.45 ± 30.72 offspring/individuals in EO and NEEO treatments. Intrinsic rate of increase (r) and finite rate of increase (*λ*) remained statistically unchanged, ranging from 0.168 to 0.176 d^−1^ and 1.18–1.19 d^−1^, respectively. Mean generation time (*T*) was significantly reduced from 33.83 ± 0.35 (control) to 28.69 ± 0.65 (LC_30_) and 27.95 ± 0.43 (LC_30_ nano) (Table 4).

### 2.5. Antioxidant Enzyme Activity

Treatments had a significant impact on antioxidant enzyme activities in second instar *S. frugiperda* larvae (Table 5). CAT and SOD activities increased with both concentration and formulation type. CAT activity was highest in the LC_50_ NEEO group (0.669 ± 0.009), followed by the LC_30_ NEEO (0.615 ± 0.002), LC_50_ EO (0.578 ± 0.003), and LC_30_ EO (0.553 ± 0.003), all significantly above the control (0.503 ± 0.005) (F = 127.66; *df* = 4,14; *p* < 0.0001). Similarly, SOD activity peaked in the LC_50_ NEEO (0.402 ± 0.009), followed by the LC_30_ NEEO (0.371 ± 0.002), LC_50_ EO (0.342 ± 0.003), and LC_30_ EO (0.300 ± 0.003), exceeding the control (0.282 ± 0.005) (F = 79.11; *df* = 4,14; *p* < 0.0001).

### 2.6. Detoxifying Enzymes Activity

The activities of detoxifying enzymes, α-NE, β-NE, GST, and CYP450, varied significantly among treatments in the second instar larvae of *S. frugiperda* (Table 6). The α-NE and β-NE activities showed a dose- and formulation-dependent decrease, with the highest value in the control (0.209 ± 0.008 and 0.350 ± 0.008, respectively) and the lowest in the LC_50_ NEEO (0.124 ± 0.003 and 0.181 ± 0.015). In contrast, GST activity increased significantly with treatment intensity. The highest GST activity with DCNB as substrate was observed in the LC_50_ NEEO (0.304 ± 0.001), and the lowest in the control (0.142 ± 0.016) (F = 40.59, *df* = 4,14; *p* < 0.0001), whereas the highest GST activity with CDNB was recorded in the LC_50_ NEEO (0.433 ± 0.010) and the lowest in the control (0.177 ± 0.012) (F = 29.93, *df* = 4,14; *p* < 0.0001). CYP450 activity followed a pattern similar to that of the esterases, with the highest value observed in the control group (0.101 ± 0.008) and the lowest in larvae exposed to the LC_50_ NEEO (0.041 ± 0.003) (F = 22.04, *df* = 4,14; *p* < 0.0001).

### 2.7. Acetylcholinesterase Activity

**Figure 5:**
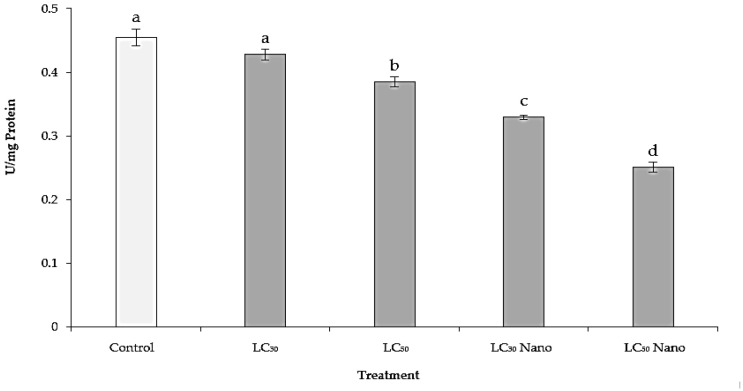
The activity of Acetylcholinesterase in second instar larvae of *Spodoptera frugiperda* was assessed 24 h post-treatment with LC_30_ and LC_50_ of *Satureja hortensis* essential oil and LC_30_ and LC_50_ of prepared nanoemulsion, along with a control group. Similar letters indicate no statistically significant differences (mean ± SE) (Tukey test, *p* ≤ 0.05).

AChE activity in *S. frugiperda* larvae was significantly inhibited by both EOs and NEEOs in comparison with the control (Figure 5). The highest AChE activity was found in the control group (0.454 ± 0.013), followed by larvae exposed to the LC_30_ (0.428 ± 0.009) and LC_50_ (0.385 ± 0.007) of the EO. The NEEO induced a more pronounced inhibition, with AChE activity declining to 0.329 ± 0.003 at the LC_30_ and further to 0.251 ± 0.008 at the LC_50_ values (F = 87.1; *df* = 4,14; *p* < 0.0001).

### 2.8. Na^+^/K^+^-ATPase Activity

**Figure 6:**
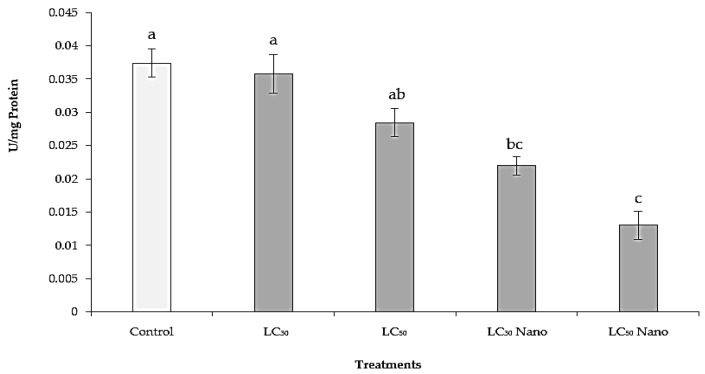
The activity of Na^+^/K^+^-ATPases in second instar larvae of *Spodopetra frugiperda* was assessed 24 h post-treatment with LC_30_ and LC_50_ of *Satureja hortensis* essential oil and LC_30_ and LC_50_ of prepared nanoemulsion, along with a control group. Similar letters indicate no statistically significant differences (mean ± SE) (Tukey test, *p* ≤ 0.05).

Exposure to both EOs and NEEOs significantly inhibited the activity of Na^+^/K^+^-ATPase in *S. frugiperda* larvae (Figure 6). The control exhibited the highest activity (0.0374 ± 0.0022), which was statistically comparable to that observed at LC_30_ of the EO (0.0358 ± 0.0029). A moderate decrease occurred at LC_50_ of the EO (0.0285 ± 0.0022), whereas the NEEO produced substantially greater inhibition, reducing activity to 0.0220 ± 0.0014 (LC_30_) and 0.0130 ± 0.0022 (LC_50_). Treatment groups differed significantly (*p* < 0.05), with LC_50_ of the NEEO causing the highest suppression (F = 20.85; *df* = 4,14; *p* < 0.0001).

## 3. Discussion

EOs are among the most promising plant-derived alternatives for sustainable insect pest management. In recent years, growing scientific interest has focused on evaluating their insecticidal potential, as EOs are environmentally friendly, biodegradable, and exhibit low toxicity to mammals [32,33]. Studies on insect pest control using EOs have revealed multiple mechanisms against target species [34]. However, practical challenges such as high volatility and instability have limited their large-scale application. Nanotechnology provides effective solutions to these issues, improving the efficiency and field applicability of eco-friendly insecticides. Advanced formulations, including NEEOs and encapsulation, enhance stability, enable controlled release, and increase efficacy while reducing required doses and environmental impact [16].

The leaves of *S. hortensis* are rich in diverse secondary metabolites with insecticidal activity, which can affect or alter insect physiology, highlighting their potential as a natural source for pest management [35]. In the present study, the chemical composition of *S. hortensis* EO was predominantly characterized by carvacrol (24.0%), γ-terpinene (21.3%), and *p*-cymene (19.2%). Similar compositional profiles have been reported in previous studies on *S. hortensis* EO, where these monoterpenoid compounds were identified as the major constituents [36,37]. For instance, Ilić et al. [38] reported that the EO of *S. hortensis* was mainly composed of carvacrol (48.7–53.25%), γ-terpinene (32.7–36.8%), *p*-cymene (3.2–3.4%), α-terpinene (2.9–3.5%), and limonene (1.3–1.9%). Similarly, Sefidkon et al. [39] found that *S. hortensis* EO predominantly contained carvacrol (56.1%) and *p*-cymene (14%). Other studies have also identified carvacrol, *p*-cymene, and γ-terpinene as the predominant constituents of *S. hortensis* EO [40,41]. Furthermore, analysis of 30 *S. hortensis* EO samples collected from different regions of Iran revealed two major chemical profiles: one characterized by carvacrol (42.0–58.2%), γ-terpinene (18.3–28.5%), and *p*-cymene (4.3–14.9%), and another strongly dominated by carvacrol (76.0–83.3%) [42].

These compounds are known to exhibit considerable biological activity against insect pests. Their insecticidal properties have been associated with the disruption of membrane permeability, interference with respiratory activity, and alterations in neurophysiological processes in insects [43,44]. Such physiological disturbances may contribute to both the lethal and sublethal effects observed following exposure to EO [45,46]. Variations in the relative abundance of EO constituents among different studies have frequently been reported and may be attributed to several factors, including geographical origin, environmental conditions (such as temperature, soil characteristics, and ecological stress), plant developmental stage, cultivation practices, and extraction procedures [47,48]. These variations can influence the biological efficacy of EOs against insect pests. Nevertheless, the predominance of bioactive monoterpenes in *S. hortensis* EO highlights its potential as a natural source of insecticidal compounds and supports its possible application in environmentally sustainable pest management programs [49].

Nanotechnology is an effective approach developed for protecting EOs from environmental impacts while improving their stability and bioavailability. This technique is beneficial in the preparation of NEEOs, where small oil droplets in the internal phase (approximately 20–1000 nm) enable effective dispersion of water-insoluble compounds, such as EOs [50]. Low-energy methods produce smaller droplets with better homogeneity than conventional approaches, allowing uniform spraying onto target surfaces and enhancing bioavailability and efficacy [51]. Recent studies have demonstrated that NEEO formulations of EOs can effectively enhance their stability and bioactivity [52,53,54]. The technique employed in this study is regarded as a “green technology” due to their high efficiency in ultrasonic homogenization and minimal equipment requirements. This approach uses less energy and surfactant while producing more uniform droplets that are smaller than those achieved with traditional mechanical methods [55,56]. In the present study, a NEEO of *S. hortensis* EO was successfully prepared using an oil-in-water emulsification method and characterized by SEM and TEM analyses. The obtained droplets were uniformly spherical, amorphous in structure, and smooth-surfaced, with an average diameter of approximately 215 nm. This droplet size falls within the typical nanometric range reported for EO NEEOs. For instance, NEEOs of *Jasminum azoricum* L. and *Mentha arvensis* L. have been reported to produce spherical particles with average diameters of approximately 199 nm and 333 nm, respectively [57]. Similarly, Tween 80-stabilized NEEOs of *Eucalyptus globulus* Labill. EO formed uniform spherical droplets with a mean diameter of 182.21 ± 35.68 nm, which contributed to improved physical stability and enhanced penetration through the insect cuticle [58]. Comparable morphological features have also been reported for plant EO NEEOs produced by ultrasonication methods, where reduced droplet size and homogeneous dispersion were associated with improved stability, increased surface area, and enhanced biological performance of the encapsulated compounds [59,60]. Moreover, previous studies have demonstrated that reducing droplet size to the nanometric scale facilitates the penetration and bioavailability of hydrophobic EO constituents in insect tissues, thereby increasing their insecticidal activity [61]. Therefore, the enhanced biological performance observed for the NEEO in the present study may be attributed, at least in part, to its favorable physicochemical characteristics, particularly its small droplet size and uniform morphology. Overall, these findings are consistent with previous reports and further confirm that nanoemulsification is an effective approach for overcoming the inherent limitations of EOs and improving their applicability in pest management strategies [62].

Previous studies have demonstrated the insecticidal activity of *S. hortensis* EO against various insect pests [22,63]. The results clearly indicate that the NEEO formulation enhanced the insecticidal activity of *S. hortensis* EO against second-instar *S. frugiperda* larvae. Specifically, the LC_50_ value of the NEEO (0.922%) was lower than that of the crude oil (1.186%), corresponding to a 1.28-fold increase in toxicity. Moreover, mortality in both treatments followed a concentration-dependent trend, while the NEEO consistently produced greater larvicidal activity than the non-formulated oil. The insecticidal activity of the EO may be associated with the presence of several bioactive constituents, particularly carvacrol, which has been reported as the predominant compound exhibiting strong toxicity against a wide range of insect pests [64,65]. In the NEEO, the insecticidal performance of carvacrol may be further enhanced through synergistic interactions with other phytochemical constituents in combination with the reduced droplet size of the formulation [66,67,68]. Moreover, the increased toxicity of the NEEO may be related to its improved dispersion and physicochemical stability, as well as the enhanced penetration and delivery of active compounds through the insect cuticle and digestive tract. Similar findings have been reported in previous studies, where nano-formulated EOs showed significantly higher toxicity against insect pests compared with bulk EOs [69,70]. For example, nanoencapsulation of botanical insecticides has been shown to increase the persistence and bioavailability of volatile terpenoid compounds, resulting in enhanced larvicidal activity and reduced effective concentrations [71]. In addition, the NEEO of lemon peel EO was also more active than the pure EO against *Agrotis ipsilon* (Hüfnagel), achieving 90% larval mortality at a concentration of 75 mg/mL [72]. Likewise, a NEEO of *Allium sativum* L. EO showed improved larvicidal activity against *S. littoralis* (Boisduval) with a LC_50_ of 12.5 µL/mL, effectively reducing larval feeding [73]. Furthermore, nanoencapsulation of *Piper aduncum* L. EO significantly enhanced its insecticidal activity against *S. frugiperda* compared to the unformulated EO, while reducing phytotoxicity and increasing stability [74]. Overall, the present findings demonstrate that NEEOs significantly enhance the insecticidal performance of plant EOs. The enhanced insecticidal activity observed for the NEEO formulation can be attributed to several physicochemical properties of NEEOs. The substantially reduced droplet size leads to a larger interfacial surface area and improved dispersion stability, which can facilitate closer interaction between the active compounds and the insect cuticle. These characteristics may enhance the penetration and bioavailability of hydrophobic EO constituents within insect tissues. Furthermore, NEEOs can improve the transport and distribution of bioactive molecules across biological barriers, thereby increasing their toxicological impact on target organisms. Such improved delivery and penetration of active compounds has been widely reported as one of the main factors responsible for the enhanced bioactivity of nanoformulated botanical insecticides [70,75].

Although the NEEO exhibited high bioactivity and satisfactory dispersion properties during the experimental period, the long-term storage stability of the formulation was not evaluated in the present study. Storage stability is an important factor influencing the practical applicability and shelf life of nanoformulated pesticides. Therefore, further studies are required to investigate the physicochemical stability of the NEEO under different storage conditions and over extended periods to ensure its suitability for large-scale application [60].

Reproductive traits such as *r*, *R*_0_, *λ*, and *GRR* are essential for explaining how nutrition affects the fitness and reproductive capacity of insect pests [76]. Studies have shown that the type and quality of nutrition impact insect reproductive performance; consequently, inadequate nutrition may lead to stunted development, reduced fecundity, and diminished reproductive potential in insect populations [77,78]. Although high mortality in pest populations is often a primary goal in pest management, it should not be regarded as the sole objective of botanical insecticide applications [14], since achieving such effects typically requires higher doses and substantial biomass. Therefore, sublethal effects on the pest’s life cycle including oviposition, reproduction, and demographic parameters are also highly relevant and desirable within integrated pest management (IPM) strategies [79]. In the present study, both the LC_30_ of the *S. hortensis* EO and its NEEO significantly suppressed the development, preadult survival, adult longevity, and reproductive performance of *S. frugiperda*, with the NEEO exhibiting stronger effects. Specifically, larval and preadult durations, fecundity, and oviposition period were more reduced following nano treatment. These findings are consistent with previous studies indicating that plant EOs, owing to the presence of compounds such as α-pinene, limonene, and terpinen-4-ol, are capable of inhibiting the developmental stages of insect pests and reducing their lifespan [79,80,81,82]. For instance, the EOs of *Eugenia uniflora* and *Melaleuca armillaris* significantly reduced the mean generation time of *S. frugiperda* [83]. Nanoencapsulation of *Cymbopogon citratus* (DC.) Stapf was shown to exert more pronounced adverse effects on the developmental duration, preadult survival, and reproductive parameters of *S. frugiperda* compared to the pure EO [84]. Overall, the present findings indicate that *S. hortensis* EO, particularly its NEEOs, has strong potential to suppress *S. frugiperda* life-history parameters by targeting multiple biological traits.

Detoxification enzymes such as CarEs, GSTs, and CYPs break down and eliminate external compounds, including pesticides and plant allelochemicals. Antioxidant enzymes like SOD and CAT reduce reactive oxygen species (ROS), which can arise during metabolic or environmental stress. Through their complementary roles, these two enzymes protect the cell by preserving homeostasis in the presence of chemical stress [85,86]. Previous studies have revealed that EOs can significantly disrupt insect physiology by inducing or suppressing the activities of detoxifying and antioxidant enzymes [87]. Based on findings of the current study, the NEEO of *S. hortensis* EO was found to increase GST, CAT, and SOD activities, along with reducing general esterase and P450 activities in *S. frugiperda* in a concentration-dependent manner compared with the pure EO. In line with our results, the EOs of *Rosmarinus officinalis* L., *Thymus vulgaris* L., and *Cymbopogon citratus* Stapf. inhibited the activity of detoxification enzymes, including cytochrome P450s, esterases, and glutathione S-transferases, in *Trichoplusia ni* Hübner larvae [88]. The NEEO of *Cymbopogon winterianus* Jowitt ex Bor enhanced CAT and GST activities in *Tribolium castaneum* Herbst more effectively than the pure EO [89]. Exposure to *Origanum majorana* L. and *Matricaria chamomilla* L. EOs and their NEEOs significantly increased GST and MFO activities in *Aphis craccivora* Koch [90]. The major terpenes present in *Satureja* species’ EOs, such as carvacrol and γ-terpinene, appear to reduce general esterase activity and inhibit cytochrome P450 monooxygenase function, thereby contributing significantly to their insecticidal effects [91,92,93]. Meanwhile, the observed increase in CAT activity is likely a regulatory response to elevated SOD activity and H_2_O_2_ levels, protecting insects against ROS-induced damage [94,95].

EOs and their components can inhibit AChE, impair neural transmission, and lead to paralysis and mortality. Additionally, these compounds may reduce Na^+^/K^+^-ATPase activity, disturbing ionic balance and cellular stability [96]. The disruption of both neural and ionic pathways generates potent toxicity and lowers the likelihood of developing resistance [97]. Our findings confirm that the EO of *S. hortensis* and its NEEO significantly inhibited AChE and Na^+^/K^+^-ATPase activities in *S. frugiperda* larvae. Notably, the inhibition of AChE and Na^+^/K^+^-ATPase enzymes by the NEEO was more effective, which corresponded with higher larval mortality. The observed inhibition of these key enzymes could be attributed to the presence of terpenic compounds recognized in the EO of *S. hortensis*, including thymol and *p*-cymene [98,99]. Moreover, the inhibition of Na^+^/K^+^-ATPase activities could make it a potential enzymatic target for its inhibition, increasing intracellular ion concentrations, promoting neuronal hyperexcitation, and ultimately leading to knockdown and mortality [100]. The bioactive components of *Artemisia lavandulaefolia* DC. EO, including β-pinene and terpinen-4-ol, significantly suppressed AChE and Na^+^/K^+^-ATPase activities in *P. xylostella* [101]. Thymol has been reported to exert significant inhibitory effects on AChE and Na^+^/K^+^-ATPase activities in *Helicoverpa armigera* (Hübner) [102]. Consistent with present findings, *O. majorana* and *Rosmarinus officinalis* L. EOs significantly inhibited AChE and Na^+^/K^+^-ATPase activities in *A. ipsilon* larvae [103]. These findings suggest that changes in the activity of such enzymes are a key mechanism underlying insecticidal effects, and it may also contribute significantly to the enhanced bioefficacy of the NEEO against target pests. Although the present study demonstrated promising insecticidal activity of the NEEO formulation under laboratory conditions, it did not include a commercial insecticide as a benchmark control. Future studies should therefore compare its efficacy with commercially available products to better assess its practical competitiveness within IPM programs.

## 4. Materials and Methods

### 4.1. Insects

*Spodoptera frugiperda* larvae and pupae were collected from maize fields around Ahvaz, Iran (31°20′–31°45′ N, 48°02′–48°40′ E), in early autumn. Larvae were reared on an artificial diet prepared according to Parra [104]. The diet included agar, ascorbic acid, brewer’s yeast, carioca beans, formaldehyde, methyl 4-hydroxybenzoate, sorbic acid, wheat germ, and a mix of phosphoric acid, propanoic acid, and water to keep microbes at bay. Diet preparation process started by boiling the beans in water, then the combined equal parts of that bean broth and distilled water to dissolve the agar for the final mixture. Adults were given a 10% honey solution. The rearing was carried out in a chamber kept at 25 ± 2 °C, with a relative humidity of 70 ± 10% and a 12:12 h photoperiod [105].

### 4.2. EO Extraction

Aerial parts of *S. hortensis* were gathered from farms in Varamin, Iran (35.35028° N, 51.63639° E). The plant material was dried in the shade and manually ground into a fine powder. Plant powder (50 g) was mixed with distilled water (750 mL), allowed to soak for 24 h, and subjected to hydrodistillation for 2 h using a Clevenger-type apparatus (J3230, Sina glass, Tehran, Iran) [43,106], following British Pharmacopoeia guidelines [British Pharmacopoeia Commission]. This process was repeated several times to obtain an adequate amount of EO. The obtained EO was dehydrated using Na_2_SO_4_ and kept at 4 °C.

### 4.3. Analysis of the EO

The *S. hortensis* EO was analyzed by gas chromatography-mass spectrometry (GC-MS). The instrument used was an Agilent 7890B gas chromatograph coupled to an Agilent 5977A mass spectrometric detector (Santa Clara, CA, USA). The GC column was an HP-5 ms capillary column 30 m in length, 0.25 mm in diameter, and with a thickness 0.25 µm. Helium was used as the carrier gas with a flow rate of 1.0 mL/min and a column head pressure set to 100 kPa. Retention indices were calculated using a series of *n*-alkanes (C_8_–C_20_) and the formula of van den Dool and Kratz [107]. The compounds were identified by comparison of the RI values and their mass spectral fragmentations with those found in the databases [108,109]. The percentages of the chemical components were determined based on peak integration without standardization.

### 4.4. Nanoformulation

*S. hortensis* NEEOs were formulated using Tween 80 (nonionic surfactant) and distilled water at the following proportions: 16.66% (*v*/*v*) EO, 16.66% (*v*/*v*) Tween 80, and 66.68% (*v*/*v*) distilled water. The resulting coarse emulsion was first homogenized by magnetic stirring and subsequently subjected to ultrasonication (200 W, 20 kHz, 10 min, 250 rpm) using a 13 mm diameter titanium probe (TT 13, Bandelin, Berlin, Germany). To prevent thermal degradation of the EO, the emulsion vessel was maintained below 10 °C using an ice bath (the emulsion ingredients were placed in a 100 mL container, which was submerged in a larger 200 mL ice bath during ultrasonication). Following ultrasonication, the formulations were centrifuged (5000 rpm, 10 min, 25 °C) to assess phase stability [110].

### 4.5. Characteristics of Nanoparticles

The physicochemical characterization of the synthesized NEEOs was conducted using transmission electron microscopy (TEM; Philips CM120, tungsten source, Eindhoven, The Netherlands) at an accelerating voltage of 80 kV. For TEM sample preparation, several droplets of the nanoparticle-containing emulsion were placed onto a carbon-coated copper grid to form a thin film and were subsequently stored in a dedicated grid box. Droplet morphology and emulsion stability were evaluated to confirm nanoscale dispersion according to the method reported by Sugumar [110]. For field-emission scanning electron microscopy (FE-SEM; Mira3, TESCAN, Brno, Czech Republic), the particle suspension was diluted 50-fold with double-ionized water and dried under vacuum at room temperature. Prior to analysis, the specimens were sputter-coated with a thin gold layer, and imaging was performed at an accelerating voltage of 10 kV [110].

### 4.6. Bioassays

The methodology described by de Menezes et al. [111], with slight modifications, was followed for larval bioassay. The *S. hortensis* EO was emulsified in an aqueous solution of 0.01 g/mL Tween^®^ 80 solution (0.01 mL) and diluted in distilled water (20 mL). The solution was added to the artificial diet when it was in semi-liquid phase at a temperature of 35 ± 2 °C, conditions that minimize the volatilization of EO. Final concentrations of the EO and its nanoformulation in the diet were adjusted to 0.5, 0.8, 1.5, 2.5, and 4 mg/mL, based on the mortality rates estimated between 10 and 90% in preliminary bioassays [112]. Control treatments included 20 mL of distilled water and 0.01 mL of the Tween^®^ 80 solution added to 200 mL of the artificial diet. After formulation, the diet with supplementation was allowed to solidify at room temperature. Aliquots (9 g portions) were prepared for each repetition. Newly molted second instar larvae were used in a completely randomized design, with 30 replicates per treatment. Individual larvae were confined in glass tubes (8 × 2.5 cm) containing portions of the diet (1 cm diameter × 1.5 cm height), and sealed with a cotton plug to prevent escape of the larvae.

### 4.7. Assessment of Life Table Parameters

To measure the life table parameters in *S. frugiperda*, newly emerged adults were paired and transferred to mesh cages to induce oviposition. Following a 24 h oviposition period, the adults were removed, and all deposited eggs were collected for further experimentation. Each egg was put individually into a plastic container with a treated artificial diet prepared according to standard rearing protocol [113,114]. The eggs were checked daily until hatching. Development and survival in larvae and pupae were monitored at 24 h intervals throughout the immature stages. After adult emergence, males and females were put together in oviposition chambers to evaluate their reproductive performance. Data related to pre-oviposition and oviposition periods, daily and total fecundity, total life span, and adult longevity were collected until the last individual in the cohort had died.

### 4.8. Preparation of Samples for Biochemical Tests

Second-instar larvae from each treatment and the control were used for enzyme activity assays. Each treatment was replicated three times, and each replicate consisted of 10 s instar larvae. The larvae were homogenized in phosphate buffer (pH 7.0) using a manual glass homogenizer. The homogenates were centrifuged at 13,000× *g* for 20 min at 4 °C, and the resulting supernatants were transferred to new microtubes. These supernatants were used as the enzyme source for subsequent biochemical analyses, which were performed using a microplate reader (BioTek EPOCH 2, Winooski, VT, USA).

### 4.9. Protein Quantification

The protein content was assayed using the Lowry et al. [115] method with a ready-to-use commercial kit (Ziest Chem Co., Ltd., Tehran, Iran). The enzyme extract (20 µL) was mixed with 100 µL of the reagent. After 30 min incubation at 25 °C, the absorbance was measured at 545 nm.

### 4.10. SOD

The SOD activity was evaluated using the xanthine oxidase–cytochrome c reduction method [116]. The reaction mixture contained 100 µL of xanthine oxidase, 500 µL of SOD solution in phosphate buffer (0.1 M, pH 7.0), and 10 mg of bovine serum albumin in 2 mL of buffer. After adding 60 µL of the supernatant, the incubation was carried out in the dark at 28 °C for 20 min. Enzymatic activity was quantified by measuring absorbance at 560 nm.

### 4.11. CAT

Enzymatic activity was measured following a modified protocol based on Wang et al. [117]. In brief, 50 µL of the supernatant from each treatment or control group was mixed with 500 µL of 1% (*w*/*v*) H_2_O_2_ in 50 mM phosphate buffer (pH 7.4). It was incubated at 28 ± 1 °C for 10 min, after which the reaction was stopped, and the degradation of H_2_O_2_ was determined by monitoring the absorbance at 240 nm.

### 4.12. General Esterase

General esterase activity was estimated according to van Asperen [118]. Homogenates from either treated or untreated larvae were placed into microtubes containing 1000 µL of phosphate buffer (0.1 M, pH 7.0) and 0.01% Triton X-100 and centrifuged (4 °C, 10 min). For the assay, 20 µL of each substrate (α- or β-naphthyl acetate, 10 mM) was mixed with 10 µL of Fast Blue RR salt (1 mM), 50 µL of phosphate buffer (20 mM, pH 7.0), and 10 µL of the enzyme extract. The mixtures were then incubated for 5 min at 25 °C before being read in a spectrophotometer with a wavelength of 450 nm.

### 4.13. GST

GST was measured by a modified spectrophotometric based on the method of Oppenorth et al. [119]. Each reaction mixture consisted of 40 µL of the enzyme-containing supernatant, 140 µL of phosphate buffer (0.1 M, pH 7.0), 100 µL of reduced glutathione (GSH), and 50 µL of CDNB (1-chloro-2,4-dinitrobenzene, 25 mM) and DCNB (3,4-dichloronitrobenzene, 20 mM) as substrates. The mixtures were incubated for 5 min at 25 °C. Enzyme activity was monitored by recording the absorbance at 340 nm.

### 4.14. CYP450

The activity of cytochrome P450 was assessed using the TMB oxidation method [120]. The assay was conducted in a 300 µL reaction mixture, comprising 55 µL of phosphate buffer (100 mM, pH 7.2), 55 µL of the enzyme extract, and 160 µL of TMB solution (0.5 mg/mL in methanol/acetate buffer). The reaction was started by adding 30 µL of 3% H_2_O_2_, incubating for 30 min at 25 °C, and the ensuing activity was measured spectrophotometrically at 630 nm.

### 4.15. AChE

The AChE activity was measured according to the method of Ellman et al. [121]. The 200 µL assay mixture consisted of 100 µL of sodium phosphate buffer (100 mM, pH 7.0), 50 µL of chromogenic reagent DTNB (100 mM), and 50 µL of substrate acetylthiocholine iodide (10 mM). After a preincubation period of 5 min, the reaction was initiated with the addition of 20 µL of the enzyme extract, and incubation was carried out at 25 °C for 30 min. The quantity of product generated was determined spectrophotometrically by absorbance at 405 nm.

### 4.16. Na^+^/K^+^-ATPase Assessment

Forty larvae were collected 48 h post-treatment, and each larva was weighed, cleaned, and homogenized in ice-cold Tris–HCl buffer (10 mM, pH 7.0) containing 0.32 M sucrose and 1 mM EDTA. Homogenate was centrifuged at 2500× *g* for 10 min at 4 °C. The pellet was then centrifuged at 22,000× *g* for 30 min and finally resuspended in Tris–HCl buffer (pH 7.4) containing 1 mM EDTA. It was stored at −20 °C until further analysis.

The activity of Na^+^/K^+^-ATPase was determined in three different reaction mixtures that consisted of the enzyme extract, 50 mM Tris–HCl (pH 7.0), and 5 mM MgCl_2_. The first mixture contained 150 mM NaCl and 20 mM KCl, the second contained 1 mM ouabain for determining ouabain-sensitive activity, and the third was used as a control. Each 1 mL reaction was performed in triplicate, preincubated for 5 min at 37 °C, and started with 1.5 mM ATP, followed by incubation for 15 min. Reactions were stopped with 15% ice-cold TCA. The inorganic phosphate was measured by reaction with 1% freshly prepared ascorbic acid and 1% ammonium molybdate in 0.5 N H_2_SO_4_. The absorbance was read at 625 nm after 30 min at 25 °C [122].

### 4.17. Data Analysis

Probit analysis, using the PoloPlus software (Version 2.0), was carried out to estimate lethal concentrations, LC_10_, LC_30_, and LC_50_, as well as their 95% CIs. The population parameters of intrinsic rate of increase (*r*), net reproductive rate (*R*_0_), finite rate of increase (*λ*), and mean generation time (*T*) were estimated using an age-stage, two-sex life table theory through the TWOSEX-MSChart program [113,114]. Standard errors for biological traits, such as fecundity, reproductive period, and developmental duration, and for population parameters were calculated using a bootstrap technique with 100,000 resamples to ensure stable and reliable results. Mean comparisons between treatments were performed using paired bootstrap tests. All bootstrap and paired bootstrap procedures were performed within the TWOSEX-MSChart software (Version 25/06/2023). Enzymatic activity data were arcsine square root transformed whenever necessary and then subjected to one-way analysis of variance (ANOVA). The means of treatment were separated using Tukey’s test (*p* < 0.05).

## 5. Conclusions

This study demonstrates that *S. hortensis* EO and its NEEO form exert strong lethal and sublethal effects on *S. frugiperda*. The NEEO increased the lethality of insect pests, reduced developmental periods, survival, fecundity, and population growth, and imposed stronger biochemical alterations, including elevated antioxidant activity and inhibition of detoxification enzymes, AChE, and Na^+^/K^+^-ATPase. It was found that the nanoemulsification enhanced the insecticidal potential of *S. hortensis* EO as a botanical tool for managing *S. frugiperda*. The limitations of the present study, including it being conducted under controlled laboratory conditions, its focus on larval stages, and its lack of non-target effects assessments, should be considered. Accordingly, future studies should validate these results under field-scale crop systems, non-target organism safety, and explore improved formulations or combinations with other biological and botanical control agents to strengthen their role in integrated pest management programs.

## Figures and Tables

**Table 1:** Chemical composition (percentages) of *Satureja hortensis* essential oil.

RI_calc_	RI_db_	Compounds	%
919	924	α-Thujene	2.2
930	932	α-Pinene	6.0
952	946	Camphene	0.1
973	974	β-Pinene	1.4
987	988	Myrcene	2.3
999	1002	α-Phellandrene	0.3
1011	1014	α-Terpinene	1.5
1026	1025	*p*-Cymene	19.2
1030	1028	Limonene	0.2
1061	1054	γ-Terpinene	21.3
1066	1065	*cis*-Sabinene hydrate	0.1
1085	1086	Terpinolene	0.2
1086	1089	*p*-Cymenene	0.2
1095	1098	*trans-*Sabinene hydrate	0.3
1125	1128	*allo*-Ocimene	0.1
1163	1162	(*E*,*E*)-2,6-Dimethyl-3,5,7-octatrien-2-ol	0.5
1175	1174	Terpinen-4-ol	0.4
1188	1186	α-Terpineol	0.1
1242	1195	Methyl chavicol (=Estragole)	0.3
1242	1241	Carvacryl methyl ether	1.0
1280	1282	(*E*)-Anethole	2.1
1289	1289	Thymol	3.3
1305	1298	Carvacrol	24.0
1314	1322	2-Methyl-5-(propan-2-ylidene)cyclohexane-1,4-diol	0.7
1367	1370	Carvacryl acetate	0.8
1375	1379	Geranyl acetate	0.1
1418	1417	(*E*)-β-Caryophyllene	0.7
1437	1439	Aromadendrene	0.3
1473	1469	β-Acoradiene	0.3
1493	1496	Viridiflorene	0.1
1504	1505	β-Bisabolene	1.7
1537	1541	(*E*)-α-Bisabolene	0.2
1558	1561	(*E*)-Nerolidol	0.1
1579	1577	Spathulenol	0.5
1586	1582	Caryophyllene oxide	1.0
1673	1668	14-Hydroxy-9-*epi*-(*E*)-caryophyllene	0.2
1842	1841	Phytone	0.2
2007	2015	13-*epi*-Manoyl oxide	0.1
		Monoterpene hydrocarbons	55.1
		Oxygenated monoterpenoids	31.3
		Sesquiterpene hydrocarbons	3.3
		Oxygenated sesquiterpenoids	1.8
		Diterpenoids	0.1
		Phenylpropanoids	2.4
		Others	2.1
		Total unidentified	96.1

RI_calc_ = retention index calculated with respect to a homologous series of *n*-alkanes using an HP-5 ms column. R_Idb_ = reference retention index from the databases.

**Table 2:** Susceptibility of *Spodoptera frugiperda* second-instar larvae to *Satureja hortensis* essential oil (EO) and its nanoemulsion (NEEO) 48 h after treatment.

Treatment.	LC_30_	LC_50_	LC_90_	*χ*^2^ (*df* = 3)	*p*	RMP
EO	0.743 (0.531–0.932)	1.186 (0.948–1.457)	3.721 (2.757–6.141)	1.974	0.658	-
NEEO	0.577 (0.386–0.742)	0.922 (0.711–1.135)	2.898 (2.182–4.668)	2.281	0.760	1.286

LC: lethal concentration; χ^2^: Chi-square value; *df*: degrees of freedom; RMP: relative median potency = the highest LC_50_ value/another LC_50_ value.

**Table 3:** Biological parameters of *Spodoptera frugiperda* exposed to *Satureja hortensis* essential oil (EO) and its nanoemulsion (NEEO) compared to the control group.

Parameter	Control		LC_30_ of EO		LC_30_ of NEEO	
	N	Mean ± SE	N	Mean ± SE	N	Mean ± SE
Egg (d)	40	2.26 ± 0.07 a	40	2.18 ± 0.06 a	40	1.87 ± 0.05 b
1st instar larvae (d)	39	2.86 ± 0.11 a	39	2.03 ± 0.16 b	39	1.89 ± 0.14 b
2nd instar larvae (d)	35	2.12 ± 0.12 a	35	1.58 ± 0.011 b	37	1.48 ± 0.09 b
3rd instar larvae (d)	34	2.15 ± 0.10 a	31	1.79 ± 0.10 b	33	1.87 ± 0.07 b
4th instar larvae (d)	34	2.18 ± 0.07 a	29	2.07 ± 0.06 a	31	2.07 ± 0.07 a
5th instar larvae (d)	34	2.97 ± 0.12 a	29	2.62 ± 0.12 b	28	2.29 ± 0.08 c
6th instar larvae (d)	34	4.44 ± 0.11 a	29	4.00 ± 0.12 b	23	3.96 ± 0.14 b
Prepupation (d)	34	1.71 ± 0.08 a	29	1.66 ± 0.09 a	23	1.57 ± 0.10 a
Pupation (d)	34	9.03 ± 0.11 a	29	7.48 ± 0.09 b	23	7.52 ± 0.10 b
Preadult (d)	34	29.74 ± 0.26 a	29	25.21 ± 0.45 a	23	24.26 ± 0.41 b
Preadult survival rate	34	0.85 ± 0.05 a	29	0.72 ± 0.07 b	23	0.57 ± 0.07 c
Female longevity (d)	18	9.56 ± 0.12 a	14	8.07 ± 0.16 b	13	7.69 ± 0.13 b
Male longevity (d)	16	8.81 ± 0.19 a	15	7.40 ± 0.13 b	10	6.70 ± 0.21 c
Total lifespan (d)	34	38.94 ± 0.31 a	29	32.93 ± 0.48 b	23	31.52 ± 0.44 b
APOP (d)	18	1.18 ± 0.07 b	14	1.42 ± 0.13 a	13	1.38 ± 0.14 a
TPOP (d)	18	30.44 ± 0.35 a	14	26.35 ± 0.65 b	13	25.54 ± 0.47 b
Fecundity (eggs/female)	18	665.56 ± 11.52 a	14	452.13 ± 18.49 b	13	410.62 ± 13.14 b
O_d_ (d)	18	6.61 ± 0.12 a	14	4.64 ± 0.19 b	13	4.38 ± 0.18 b

APOP: adult pre-oviposition period; TPOP: total pre-oviposition period. Results are shown as means (±SE) followed by different letters in each row, which signify significant differences among the treatment and control using the paired bootstrap test (B = 100,000) at 5% significance level.

**Table 4:** Population growth parameters (mean ± SE) of *Spodoptera frugiperda* exposed to *Satureja hortensis* essential oil (EO) and its nanoemulsion (NEEO), compared to the control group.

Parameter	Control	LC_30_ of EO	LC_30_ of NEEO
*GRR* (eggs/individuals)	377.45 ± 61.93 a	338.83 ± 94.82 a	2298.71 ± 67.15 a
*R*_0_ (eggs/individuals)	299.51 ± 52.59 a	158.25 ± 34.58 b	133.45 ± 30.72 b
*r* (d^−1^)	0.168 ± 0.005 a	0.176 ± 0.008 a	0.175 ± 0.009 a
*λ* (d^−1^)	1.18 ± 0.006 a	1.19 ± 0.010 a	1.19 ± 0.10 a
*T* (d)	33.83 ± 0.35 a	28.69 ± 0.65 b	27.95 ± 0.43 b

*GRR*: gross reproductive rate; *R*_0_: net reproductive rate; *r:* intrinsic rate of increase; *λ:* finite rate of increase; *T*: mean generation time. Results are shown as means (±SE) followed by different letters in each row, which signify significant differences among the treatment and control using the paired bootstrap test (B = 100,000) at 5% significance level.

**Table 5:** Antioxidant enzyme functions observed in the second developmental stage of *Spodoptera frugiperda* larvae treated by *Satureja hortensis* essential oil (EO) and its nanoemulsion (NEEO), compared to the control group.

Treatments	Antioxidant Enzymes	
	CAT	SOD
Control	0.503 ± 0.005 d	0.282 ± 0.005 d
LC_30_ of EO	0.553 ± 0.003 c	0.300 ± 0.003 d
LC_50_ of EO	0.578 ± 0.003 c	0.342 ± 0.003 c
LC_30_ of NEEO	0.615 ± 0.002 b	0.371 ± 0.002 b
LC_50_ of NEEO	0.669 ± 0.009 a	0.402 ± 0.009 a
F	127.66	79.11
*p*	0.0001	0.0001
*df*	4, 14	4, 14

CAT: catalase; SOD: superoxide dismutase; Unit: (U/mg protein). Results are shown as means (±SE), with identical letters in columns indicating no statistically significant difference (*p* < 0.05) based on the Tukey test. Each treatment consisted of three replicates, and each replicate contained 10 s-instar larvae.

**Table 6:** Detoxifying enzyme functions observed in the second developmental stage of *Spodoptera frugiperda* larvae treated with *Satureja hortensis* essential oil (EO) and its nanoemulsion (NEEO), compared to the control group.

Treatments	Detoxifying Enzymes				
	α-NE	β-NE	GST (DCNB)	GST (CDNB)	CYP450
Control	0.209 ± 0.008 a	0.350 ± 0.008 a	0.142 ± 0.016 c	0.177 ± 0.012 d	0.101 ± 0.008 a
LC_30_ of EO	0.198 ± 0.007 ab	0.346 ± 0.004 a	0.166 ± 0.008 bc	0.239 ± 0.016 cd	0.095 ± 0.003 ab
LC_50_ of EO	0.175 ± 0.003 bc	0.224 ± 0.009 bc	0.207 ± 0.008 b	0.312 ± 0.005 bc	0.070 ± 0.005 bc
LC_30_ of NEEO	0.149 ± 0.004 cd	0.262 ± 0.011 b	0.257 ± 0.010 a	0.393 ± 0.009 ab	0.053 ± 0.004 cd
LC_50_ of NEEO	0.124 ± 0.003 d	0.181 ± 0.015 c	0.304 ± 0.001 a	0.433 ± 0.010 a	0.041 ± 0.003 d
F	37.87	50.48	40.59	29.93	22.04
*p*	0.0001	0.0001	0.0001	0.0001	0.0001
*df*	4, 14	4, 14	4, 14	4, 14	4, 14

α-NE: α-naphtyl esterase; β-NE: β-naphtyl esterase; GST: glutathione S-transferase; CYP450: cytochrome P450; Unit: (ΔOD/min/mg protein). Results are shown as means (±SE), with identical letters in columns indicating no statistically significant difference (*p* < 0.05) based on the Tukey test. Each treatment consisted of three replicates, and each replicate contained 10 s-instar larvae.

## Data Availability

The data presented are available on request from the corresponding authors.
